# Photobiomodulation with near infrared light mitigates Alzheimer’s disease-related pathology in cerebral cortex – evidence from two transgenic mouse models

**DOI:** 10.1186/alzrt232

**Published:** 2014-01-03

**Authors:** Sivaraman Purushothuman, Daniel M Johnstone, Charith Nandasena, John Mitrofanis, Jonathan Stone

**Affiliations:** 1 Bosch Institute, University of Sydney NSW 2006, Australia; 2 Discipline of Physiology, Anderson Stuart Building F13, University of Sydney NSW 2006, Australia; 3 Discipline of Anatomy & Histology, Anderson Stuart Building F13, University of Sydney NSW 2006, Australia

## Abstract

**Introduction:**

Previous work has demonstrated the efficacy of irradiating tissue with red to infrared light in mitigating cerebral pathology and degeneration in animal models of stroke, traumatic brain injury, parkinsonism and Alzheimer’s disease (AD). Using mouse models, we explored the neuroprotective effect of near infrared light (NIr) treatment, delivered at an age when substantial pathology is already present in the cerebral cortex.

**Methods:**

We studied two mouse models with AD-related pathologies: the K369I tau transgenic model (K3), engineered to develop neurofibrillary tangles, and the APPswe/PSEN1dE9 transgenic model (APP/PS1), engineered to develop amyloid plaques. Mice were treated with NIr 20 times over a four-week period and histochemistry was used to quantify AD-related pathological hallmarks and other markers of cell damage in the neocortex and hippocampus.

**Results:**

In the K3 mice, NIr treatment was associated with a reduction in hyperphosphorylated tau, neurofibrillary tangles and oxidative stress markers (4-hydroxynonenal and 8-hydroxy-2′-deoxyguanosine) to near wildtype levels in the neocortex and hippocampus, and with a restoration of expression of the mitochondrial marker cytochrome c oxidase in surviving neurons. In the APP/PS1 mice, NIr treatment was associated with a reduction in the size and number of amyloid-β plaques in the neocortex and hippocampus.

**Conclusions:**

Our results, in two transgenic mouse models, suggest that NIr may have potential as an effective, minimally-invasive intervention for mitigating, and even reversing, progressive cerebral degenerations.

## Introduction

Alzheimer’s disease (AD) is a chronic, debilitating neurodegenerative disease with limited therapeutic options; at present there are no treatments that prevent the physical deterioration of the brain and the consequent cognitive deficits. Histopathologically, AD is characterised by neurofibrillary tangles (NFTs) of hyperphosphorylated tau protein and amyloid-beta (Aβ) plaques [1,2]. The extent of these histopathological features is considered to vary with and to determine clinical disease severity [2]. While the initiating pathogenic events underlying AD are still debated, there is strong evidence to suggest that oxidative stress and mitochondrial dysfunction have important roles in the neurodegenerative cascade [3-5]. Therefore, it has been proposed that targeting mitochondrial dysfunction could prove valuable for AD therapeutics [6].

One safe, simple yet effective approach to the repair of damaged mitochondria is photobiomodulation with near-infrared light (NIr). This treatment, which involves the irradiation of tissue with low intensity light in the red to near-infrared wavelength range (600 to 1000 nm), was originally pioneered for the healing of superficial wounds [7] but has been recently shown to have efficacy in protecting the central nervous system. While the mechanism of action remains to be elucidated, there is evidence that NIr preserves and restores cellular function by reversing dysfunctional mitochondrial cytochrome *c* oxidase (COX) activity, thereby mitigating the production of reactive oxygen species and restoring ATP production to normal levels [8,9].

To date, NIr treatment has yielded neuroprotective outcomes in animal models of retinal damage [9,10], traumatic brain injury [11,12], Parkinson’s disease [13-15] and AD [16,17]. Furthermore, NIr therapy has yielded beneficial outcomes in clinical trials of human patients with mild to moderate stroke [18] and depression [19]. This treatment represents a promising alternative to drug therapy because it is safe, easy to apply and has no known side-effects at levels even higher than optimal doses [20].

The aim of this study was to assess the efficacy of NIr in mitigating the brain pathology and associated cellular damage that characterise AD. We utilised two mouse models, each manifesting distinct AD-related pathologies: the K3 tau transgenic model, which develops NFTs [21,22]; and the APP/PS1 transgenic model, which develops amyloid plaques [23]. Here, we present histochemical evidence that NIr treatment over a period of 1 month reduces the severity of AD-related pathology and oxidative stress and restores mitochondrial function in brain regions susceptible to neurodegeneration in AD, specifically the neocortex and hippocampus. The findings extend our previous NIr work in models of acute neurodegeneration [13,14] to demonstrate that NIr is also effective in protecting the brain against chronic insults due to AD-related genetic aberrations, a pathogenic mechanism that is likely to more closely model the human neurodegenerative condition.

## Methods

### Mouse models

The K3 transgenic mouse model, originally generated as a model of frontotemporal dementia [21,22], harbours a human tau gene with the pathogenic K369I mutation; expression is driven by the neuron-specific mThy1.2 promoter. This model manifests high levels of hyperphosphorylated tau and NFTs by 2 to 3 months of age and cognitive deficits by about 4 months of age [21,22]. We commenced our experiments on K3 mice and matched C57BL/6 wildtype (WT) controls at 5 months of age, when significant neuropathology is already present.

The APPswe/PSEN1dE9 (APP/PS1) transgenic mouse model, obtained from the Jackson Laboratory (Stock number 004462; Bar Harbor, ME, USA), harbours two human transgenes: the amyloid beta precursor protein gene (APP) containing the Swedish mutation; and the presenilin-1 gene (PS1) containing a deletion of exon 9 [23]. The APP/PS1 mice exhibit increased Aβ and amyloid plaques by 4 months of age [24] and cognitive deficits by 6 months of age [25]. We commenced our experiments on APP/PS1 mice and matched C57BL/6 × C3H WT controls at 7 months of age, when numerous amyloid plaques and associated cognitive deficits are present.

Genotyping of mice was achieved by extracting DNA from tail tips through a modified version of the Hot Shot preparation method [26] and amplifying the transgene sequence by polymerase chain reaction. As reported previously, K3 mice were identified using the primers 5′-GGGTGTCTCCAATGCCTGCTTCTTCAG-3′ (forward) and 5′-AAGTCACCCAGCAGGGAGGTGCTCAG-3′ (reverse) [21,22] and APP/PS1 mice were genotyped using primers 5′-AGGACTGACCACTCGACCAG-3′ (forward) and 5′-CGGGGGTCTAGTTCTGCAT-3′ (reverse) [23].

### Experimental design

For each series of experiments on K3 mice (aged 5 months) or APP/PS1 mice (aged 7 months) there were three experimental groups: untreated WT mice, untreated transgenic mice and NIr-treated transgenic mice (*n* = 5 mice per experimental group for the K3 series, 15 mice in total; *n* = 6 mice per experimental group for the APP/PS1 series, 18 mice in total). Our design did not include a WT control group exposed to NIr because NIr has no detectable impact on the survival and function of cells in normal healthy brain [13-15]. Given the consistency of the previous results, use of animals for this extra control group did not seem justified [27].

Mice in the NIr-treated groups were exposed to one 90-second cycle of NIr (670 nm) from a light-emitting device (LED) (WARP 10; Quantum Devices, Barneveld, WI, USA) for 5 days per week over 4 consecutive weeks. Light energy emitted from the LED during each 90-second treatment equates to 4 Joule/cm^2^; a total of 80 Joule/cm^2^ was delivered to the skull over the 4 weeks. Our measurements of NIr penetration across the fur and skull of a C57BL/6 mouse indicate that ~2.5% of transmitted light reaches the cortex.

For each treatment, the mouse was restrained by hand and the LED was held 1 to 2 cm above the head. The LED light generated no heat and reliable delivery of the radiation was achieved [13-15]. For the sham-treated WT, K3 and APP/PS1 groups, animals were restrained in the same way and the device was held over the head, but the light was not switched on. This treatment regime is similar to that used in previous studies where beneficial changes to neuropathology and behavioural signs were reported [13-15].

Experimental animals were housed two or more to a cage and kept in a 12-hour light (<5 lux)/dark cycle at 22°C; food pellets and water were available *ad libitum*. All protocols were approved by the Animal Ethics Committee of the University of Sydney.

### Histology and immunohistochemistry

At the end of the experimental period, mice were anaesthetised by intraperitoneal injection of sodium pentobarbital (60 mg/kg) and perfused transcardially with 4% buffered paraformaldehyde. Brains were post fixed for 3 hours, washed with phosphate-buffered saline and cryoprotected in 30% sucrose/phosphate-buffered saline. Tissue was embedded in OCT compound (ProSciTech, Thuringowa, QLD, Australia) and coronal sections of the neocortex and the hippocampus (between bregma −1.8 and −2.1) were cut at 20 μm thickness on a Leica cryostat (Nussloch, Germany).

#### Immunohistochemistry

For most antibodies, antigen retrieval was achieved using sodium citrate buffer with 0.1% Triton. Sections were blocked in 10% normal goat serum and then incubated overnight at 4°C with a mouse monoclonal antibody – paired helical filaments-tau AT8, 1:500 (Innogenetics, Ghent, Belgium); 4-hydroxynonenal (4-HNE), 1:200 (JaICA, Fukuroi, Shizuoka, Japan); 8-hydroxy-2′-deoxyguanosine (8-OHDG), 1:200 (JaICA); COX, 1:200 (MitoSciences, Eugene, OR, USA) – and/or a rabbit polyclonal antibody (200 kDa neurofilament, 1:500; Sigma, St. Louis, MO, USA). Sections were then incubated for 3 hours at room temperature in Alexa Fluor-488 (green) and/or Alexa Fluor-594 (red) tagged secondary antibodies specific to host species of the primary antibodies (1:1,000; Molecular Probes, Carlsbad, CA, USA). Sections were then counterstained for nuclear DNA with bisbenzimide (Sigma).

Two different but complementary antibodies were used to label Aβ peptide: 6E10, which recognises residues 1 to 16; and 4G8, which recognises residues 17 to 24. We have previously used these two antibodies in combination to validate Aβ labelling, demonstrating identical labelling patterns in the rat neocortex and hippocampus [28]. For double labelling using 6E10 antibodies (1:500; Covance, Princeton, NJ, USA) and anti-glial fibrillary acidic protein antibodies (1:1,000; DAKO, Glostrup, Denmark), antigen retrieval was achieved by incubation in 90% formic acid for 10 minutes, and primary antibody incubation was carried out overnight at room temperature. For labelling using the 4G8 (1:500; Covance) antibody, slides were treated with 3% H_2_O_2_ in 50% methanol, incubated in 90% formic acid and then washed several times in dH_2_O before the blocking step, as described previously [28]. After blocking, sections were incubated overnight at room temperature with 4G8 antibody. Sections were then incubated in biotinylated goat anti-mouse IgG for 1 hour followed by ExtrAvidin peroxidase for 2.5 hours. The sections were then washed and developed with 3,3′-Diaminobenzidine.

Negative control sections were processed in the same fashion as described above except that primary antibodies were omitted. These control sections were immunonegative. Fluorescent images were taken using a Zeiss Apotome 2, Carl Zeiss, Oberkochen, Germany. Brightfield images were taken using a Nikon Eclipse E800, Nikon Instruments, Melville, NY, USA.

#### Histology

NFTs were assessed using the Bielschowsky silver staining method, as described previously [21,22]. Briefly, sections were placed in prewarmed 10% silver nitrate solution for 15 minutes, washed and then placed in ammonium silver nitrate solution at 40°C for a further 30 minutes. Sections were subsequently developed for 1 minute and then transferred to 1% ammonium hydroxide solution for 1 minute to stop the reaction. Sections were then washed in dH_2_O, placed in 5% sodium thiosulphate solution for 5 minutes, washed, cleared and mounted in dibutyl phathalate xylene.

As described previously [28], Aβ plaques were studied by staining with Congo red, a histological dye that binds preferentially to compacted amyloid with a β-sheet secondary structure [29]. Briefly, sections were treated with 2.9 M sodium chloride in 0.01 M NaOH for 20 minutes and were subsequently stained in filtered alkaline 0.2% Congo red solution for 1 hour.

### Morphological analysis

#### Staining intensity and area measurements

To quantify the average intensity and area of antibody labelling within the neocortex and hippocampal regions, an integrated morphology analysis was undertaken using MetaMorph software. For each section, the level of nonspecific staining (using an adjacent region of unstained midbrain) was adjusted to a set level to ensure a standard background across different groups. Next, outlines of retrosplenial cortex area 29 and hippocampal CA1 region were traced and the average intensity and area of immunostaining were calculated by the program. Measurements were conducted on ≥4 representative sections per animal and ≥3 animals per experimental group. Statistical analyses were performed in Prism 5.0 (Graphpad, La Jolla, CA, USA) using one-way analysis of variance with Tukey’s multiple comparison post test. All values are given as mean ± standard error of mean.

#### Amyloid-beta plaque measurements

Digital brightfield images of 4G8 staining in the neocortical and hippocampal regions (between bregma −1.8 and −2.1) were taken at 4× magnification and analysed with Metamorph, Molecular Devices LLC, Sunnyvale, CA, USA. The software was programmed to measure the number of plaques and the average size of plaques after thresholding for colour. The percentage of area covered by plaques (plaque burden) was calculated by multiplying the number of plaques by the average size of plaques, divided by the area of interest, as described previously [30]. The average number of Congo red-positive plaques in the APP/PS1 brain regions was estimated using the optical fractionator method (StereoInvestigator; MBF Science, Williston, VT, USA), as outlined previously [14]. Briefly, systematic random sampling of sites was undertaken using an unbiased counting frame (100 μm × 100 μm). All plaques that came into focus within the frame were counted. Measurements were conducted on ≥4 representative sections per animal and ≥3 animals per experimental group. Plaque numbers and size were analysed using a two-tailed unpaired *t* test (when variances were equal) or Welch’s *t* test (when variances were unequal). All values are given as mean ± standard error of mean. For all analyses, investigators were blinded to the experimental groups.

## Results

Evidence of NIr-induced neuroprotection is presented from the neocortex (retrosplenial area) and the hippocampus (CA1 and subiculum), two cortical regions affected in the early stages of human AD [2].

### Near-infrared light mitigates the tau pathology of K3 cortex

**Figure 1:**
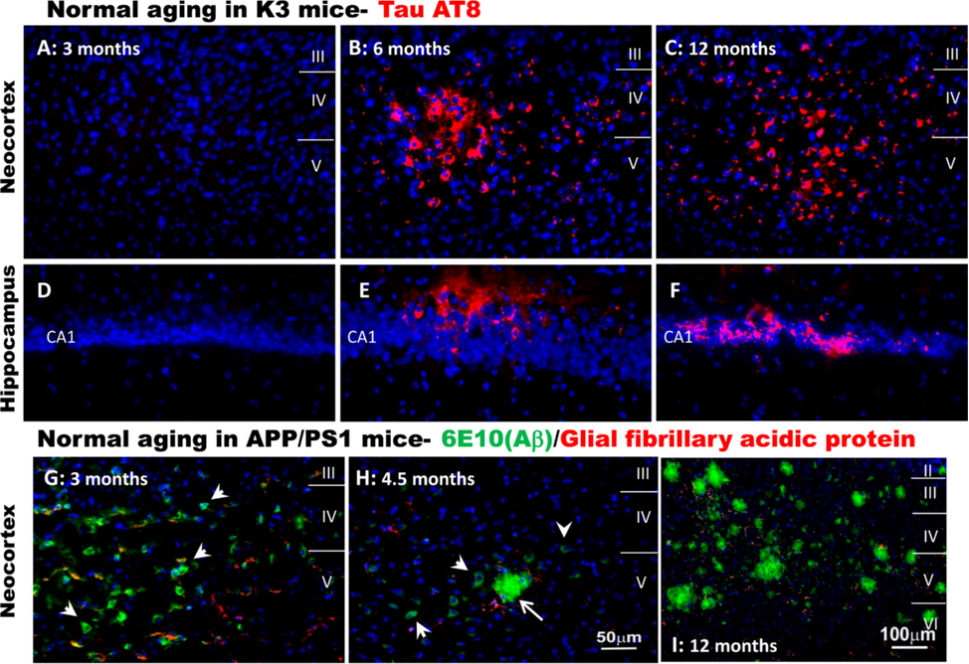
**Time course of the natural development of cortical pathology in K3 and APP/PS1 mice. (A), (B), (C), (D), (E), (F)** Micrographs of hyperphosphorylated tau labelling (red), using the AT8 antibody, in the neocortex (**A** to **C**) and hippocampus (**D** to **F**) of untreated K3 mice at 3 months **(A, D)**, 6 months **(B, E)** and 12 months **(C, F)** of age. **(G), (H), (I)** Micrographs of amyloid-beta (Aβ) labelling (green), using the 6E10 antibody, in neocortex of untreated APP/PS1 mice at 3 months **(G)**, 4.5 months **(H)** and 12 months **(I)** of age. Arrowheads indicate intraneuronal Aβ labelling, arrows indicate extracellular plaques. Comparable immunolabelling was achieved with the 4G8 antibody. Sections were co-labelled for glial fibrillary acidic protein (red), a marker of astrocytes. For all sections, nuclei were labelled with bisbenzimide (blue). Scale in **(H)** applies to **(A)** to **(G)**.

Hyperphosphorylation of the neuronal microtubule stabilising protein tau and the resulting NFTs are much studied features of dementia pathology [2,31]. The K3 mouse model manifests hyperphosphorylated tau and NFTs by 2 to 3 months of age and cognitive deficits by about 4 months of age [21,22]. We observe strong labelling for hyperphosphorylated tau in the neocortex and the hippocampus at 6 months of age; expression appears to plateau after this age, with similar labelling observed in 12-month-old mice (Figure 1A,B,C,D,E,F).

**Figure 2:**
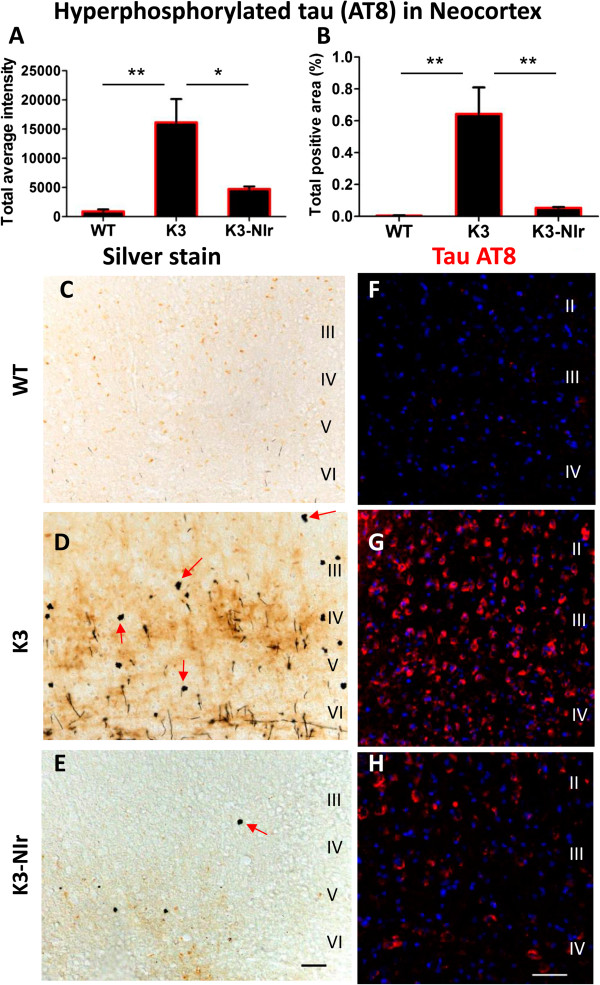
**Effect of near-infrared light treatment on hyperphosphorylated tau and neurofibrillary tangles in the neocortex of K3 mice. (A), (B)** Quantification of tau AT8 immunolabelling, based on average labelling intensity **(A)** and labelled area **(B)**. All error bars indicate standard error of mean. *P < 0.05, **P < 0.01. **(C), (D), (E)** Representative photomicrographs of sections stained with Bielschowsky silver stain to demonstrate neurofibrillary tangles (NFTs). Arrows indicate axonal swellings and NFTs. **(F), (G), (H)** Representative micrographs of AT8 (red) labelling within neurons of the neocortex retrosplenial area. Nuclei were labelled with bisbenzimide (blue). Scale bars = 50 μm; scale in **(E)** applies to **(C)** and **(D)**, scale in **(H)** applies to **(F)** and **(G)**. NIr, near-infrared light; WT, wildtype.

In the retrosplenial area of the neocortex there was a significant overall difference in AT8 immunolabelling for tau between the experimental groups, both when considering average intensity of labelling (*P* < 0.01 by analysis of variance; Figure 2A) and labelled area (*P* < 0.01; Figure 2B). Tukey *post hoc* testing revealed significant differences between the untreated K3 group and the other two groups; labelling was much stronger and more widespread in K3 mice than WT controls (17-fold higher intensity, *P* < 0.01), and this labelling was reduced by over 70% in NIr-treated mice (*P* < 0.05). Interestingly, there was no significant difference between the WT and K3-NIr groups, suggesting that NIr treatment had reduced hyperphosphorylated tau to control levels in K3 mice. A similar trend was observed when considering the NFT pathology (Figure 2C,D,E). In contrast to WT brain, which showed no NFT-like lesions (Figure 2C), the K3 brain contained many ovoid shaped NFT-like lesions (that is, spheroids; Figure 2D). Such structures were less frequent in the K3-NIr brain (Figure 2E).

**Figure 3:**
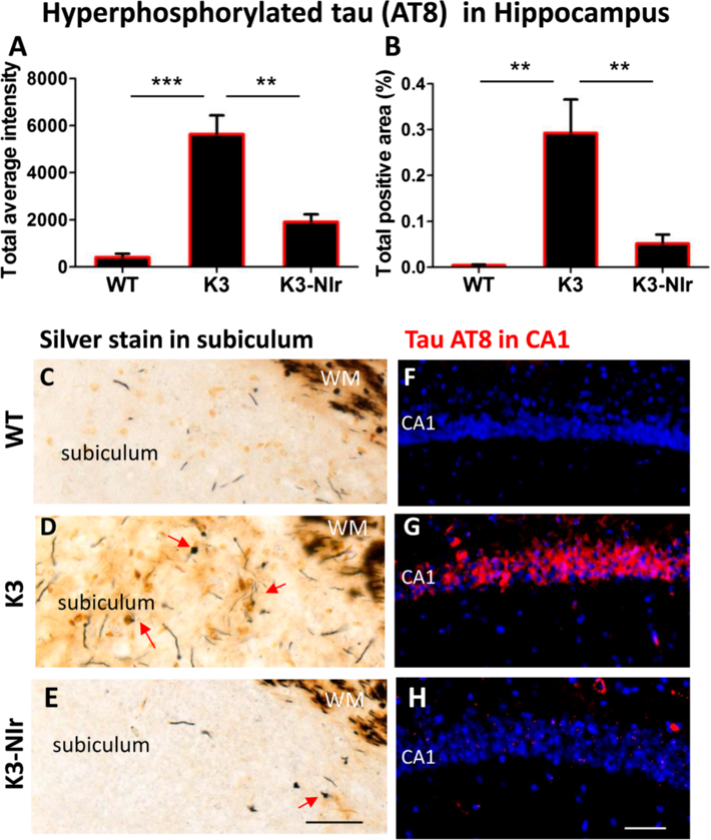
**Effect of near-infrared light treatment on hyperphosphorylated tau and neurofibrillary tangles in the hippocampus of K3 mice. (A), (B)** Quantification of tau AT8 immunolabelling, based on average labelling intensity **(A)** and labelled area **(B)**. All error bars indicate standard error of mean. **P < 0.01, ***P < 0.001. **(C), (D), (E)** Representative photomicrographs of sections of the hippocampal subiculum area, stained with Bielschowsky silver stain to demonstrate neurofibrillary tangles (NFTs). Arrows indicate axonal swellings and NFTs. The classical silver stain also labelled axons in the white matter core (WM). **(F), (G), (H)** Representative micrographs of AT8 (red) labelling within hippocampal CA1 pyramidal neurons. Nuclei were labelled with bisbenzimide (blue). Scale bars = 50 μm; scale in **(E)** applies to **(C)** and **(D)**, scale in **(H)** applies to **(F)** and **(G)**. NIr, near-infrared light; WT, wildtype.

Similar effects were observed in the hippocampus (Figure 3). There was a significant overall difference between the experimental groups in AT8 immunolabelling of the CA1 pyramidal cells (*P* < 0.01). As for the neocortex, K3 mice showed far greater labelling than WT mice (17-fold higher intensity, *P* < 0.01) and this was reduced over 65% by NIr treatment (*P* < 0.01). Again, there were no significant differences between the WT and K3-NIr groups (*P* > 0.05). Bielschowsky silver staining of the subiculum (Figure 3C,D,E) revealed axonal swellings and spheroids in the hippocampal region of K3 mice (Figure 3D), which were less pronounced in mice from the K3-NIr group (Figure 3E). No pathology was observed in the hippocampus of WT mice (Figure 3C).

One should note that the large white matter pathways associated with the hippocampus were labelled intensely by silver staining in all three groups (Figure 3C,D,E). This labelling has been described previously and is not associated with any neuropathology [32].

### Near-infrared light reduces oxidative stress in K3 cortex

Oxidative stress and damage are common features of neurodegenerative diseases such as AD, and may be a precursor to neuronal death [3-5]. We assessed two common markers of oxidative stress: 4-HNE, a toxic end-product of lipid peroxidation that may bind to proteins that then trigger mitochondrial dysfunction and cellular apoptosis in AD [33]; and 8-OHDG, a marker for nuclear and mitochondrial DNA oxidation, which is elevated in AD brains [34].

**Figure 4:**
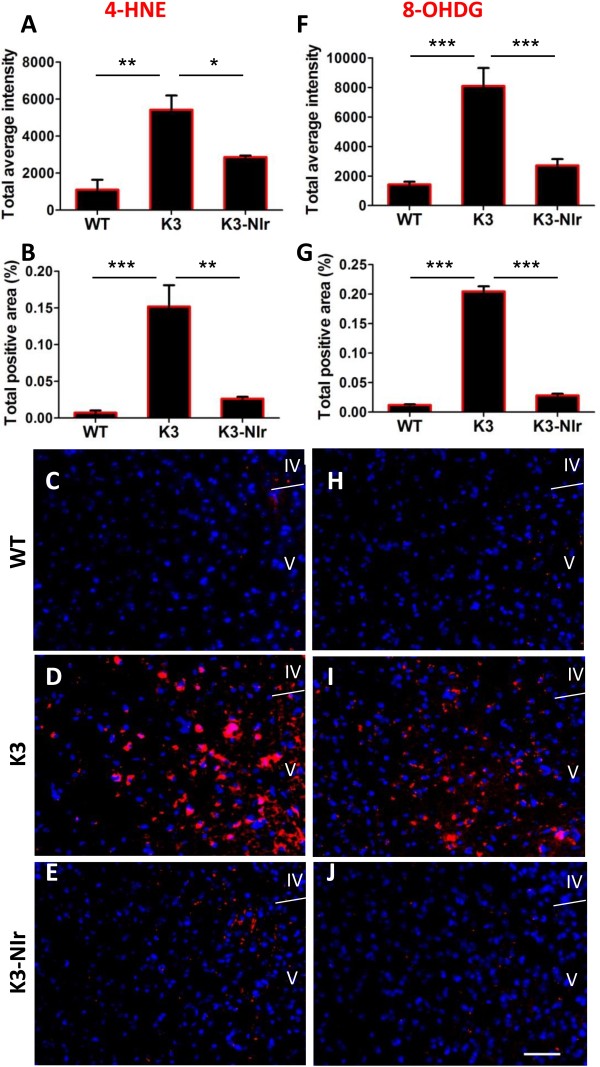
**Effect of near-infrared light treatment on oxidative stress markers in the neocortex of K3 mice. (A), (B), (F), (G)** Quantification of immunolabelling of two oxidative stress markers, 4-hydroxynonenal (4-HNE; **A, B**) and 8-hydroxy-2′-deoxyguanosine (8-OHDG; **F, G**), based on average labelling intensity **(A, F)** and labelled area **(B, G)**. All error bars indicate standard error of the mean. *P < 0.05, **P < 0.01, ***P < 0.001. **(C), (D), (E), (H), (I), (J)** Representative micrographs of 4-HNE (red) labelling **(C, D, E)** and 8-OHDG (red) labelling **(H, I, J)** within layers IV and V of the neocortical retrosplenial area. Nuclei were labelled with bisbenzimide (blue). Scale bar = 50 μm; scale in **(J)** applies to all other micrographs. NIr, near-infrared light; WT, wildtype.

Overall, 4-HNE immunoreactivity in the neocortex was significantly different between the experimental groups (Figure 4), by both average labelling intensity (*P* < 0.01) and labelled area (*P* < 0.001). As with AT8 labelling above, the K3 group showed a much higher average 4-HNE labelling intensity and area than the WT group (fivefold and 20-fold, respectively) and this labelling was significantly reduced (by 50% and 80%, respectively) in the K3-NIr group. Again, these measures showed no significant differences between the WT and K3-NIr groups (*P* > 0.05).

Similar patterns were observed for 8-OHDG immunoreactivity. Overall, there was a significant difference between the groups for 8-OHDG immunolabelling, by both average intensity (*P* < 0.0001) and labelled area (*P* < 0.0001). Again the K3 group showed significantly higher 8-OHDG labelling intensity and area than the WT group (sixfold and 17-fold, respectively), and the 8-OHDG labelling intensity and area were significantly reduced in the K3-NIr group relative to untreated K3 (65% and 85% reduction, respectively). The intensity and area of 8-OHDG labelling did not differ significantly between the WT and the K3-NIr groups (*P* > 0.05), suggesting that NIr treatment reduces markers of oxidative stress to control levels. The representative photomicrographs of 8-OHDG immunoreactivity in the retrosplenial area (Figure 4H,I,J) reflect the quantitative data, with many 8-OHDG^+^ structures in the K3 group (Figure 4I) but not in the WT and K3-NIr groups (Figure 4H,J).

### Near-infrared light mitigates mitochondrial dysfunction in K3 cortex

**Figure 5:**
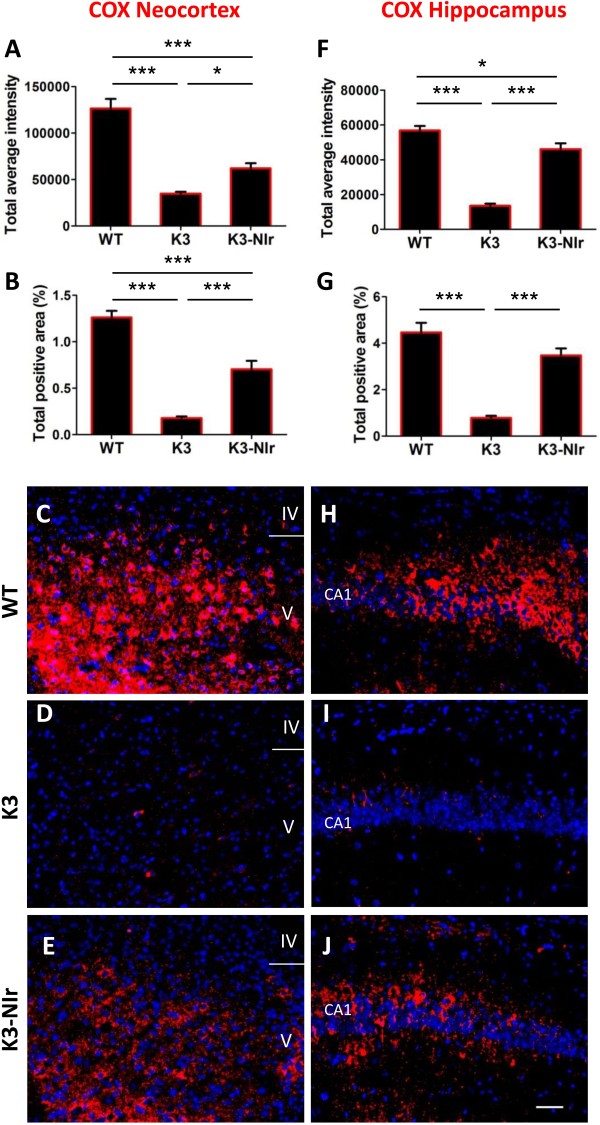
**Effect of near-infrared light treatment on cytochrome *c *oxidase labelling in the neocortex and hippocampus of K3 mice. (A), (B), (F), (G)** Quantification of immunolabelling of the mitochondrial marker cytochrome *c* oxidase (COX) in the neocortex retrosplenial area **(A, B)** and hippocampal CA1 layer (**F, G**), based on average labelling intensity **(A, F)** and labelled area **(B, G)**. All error bars indicate standard error of the mean. *P < 0.05, ***P < 0.001. **(C), (D), (E), (H), (I), (J)** Representative micrographs of COX (red) labelling in the neocortex retrosplenial area **(C, D, E)** and hippocampal CA1 layer **(H, I, J)**. Nuclei were labelled with bisbenzimide (blue). Scale bar = 50 μm; scale in **(J)** applies to all other micrographs. NIr, near-infrared light; WT, wildtype.

We assessed expression patterns of the mitochondrial enzyme COX in the neocortex and the hippocampus as a marker of mitochondrial function. Overall, there were statistically significant differences in the patterns of COX immunoreactivity between the different experimental groups, both in the neocortex and the hippocampus (both *P* < 0.0001; Figure 5). Relative to WT mice, the COX labelling intensity and area were reduced in K3 mice in both the neocortex and the hippocampus (>70% and >75% reductions, respectively). The K3-NIr mice showed a significant recovery of COX immunoreactivity relative to untreated K3 mice in both the neocortex (>1.7-fold increase, *P* < 0.05) and the hippocampus (>3.4-fold increase, *P* < 0.001). However, recovery was not complete, with K3-NIr mice having significantly lower COX immunoreactivity than WT mice in the neocortex (~50%, *P* < 0.001) and significantly lower COX labelling intensity (~20%, *P* < 0.05) in the hippocampus. These two groups did not differ significantly in COX labelling area in the hippocampus (*P* > 0.05).

### Near-infrared mitigates amyloid pathology in APP/PS1 cortex

Along with NFTs, Aβ plaques are considered a primary pathological hallmark of AD and Aβ load is often used as a marker of AD severity [1,35]. We assessed the distribution of Aβ plaques and more immature forms of the Aβ peptide in the neocortex and hippocampus of APP/PS1 mice aged 7 months; this age is after the first signs of intracellular Aβ within cells (at 3 months; Figure 1G) and extracellular Aβ plaques (at 4.5 and 12 months; Figure 1H and 1I, respectively).

**Figure 6:**
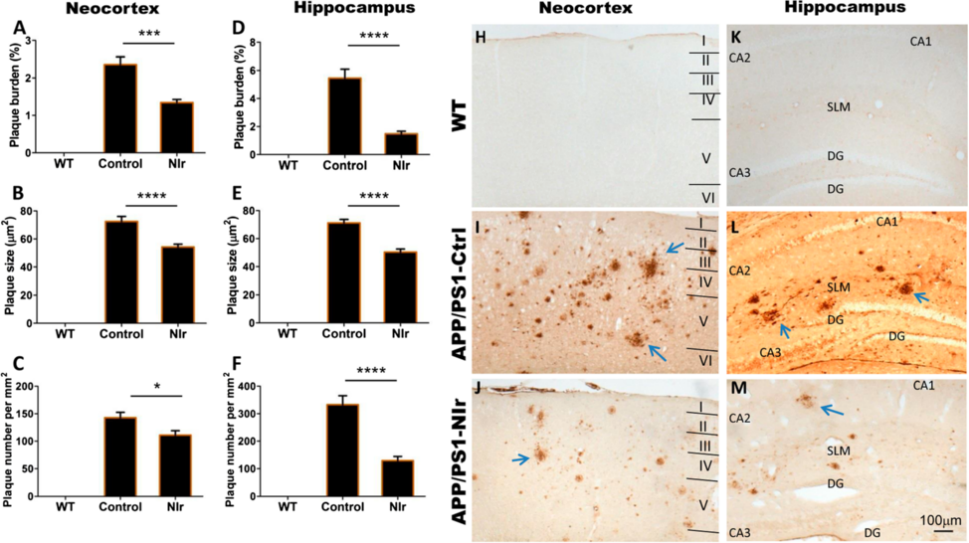
**Effect of near-infrared light on amyloid-beta and plaque pathology in APP/PS1 mice. (A), (B), (C), (D), (E), (F)** Quantification of amyloid-beta (Aβ) 4G8 immunolabelling of amyloid plaques in the neocortex **(A, B, C)** and hippocampus **(D, E, F)**, based on plaque burden **(A, D),** plaque size **(B, E)** and number of plaques **(C, F)**. All error bars indicate standard error of the mean. *P < 0.05, ***P < 0.001, ****P < 0.0001. **(H), (I), (J), (K), (L), (M)** Representative micrographs showing Aβ labelling with the 4G8 antibody (brown) in the neocortex **(H, I, J)** and hippocampus **(K, L, M)**. Arrows indicate plaques. Scale bar = 100 μm; scale in **(M)** applies to all other micrographs. DG, dentate gyrus of hippocampus; NIr, near-infrared light; SLM, stratum lacunosum moleculare; WT, wildtype.

Three quantitative measures of plaque pathology were used: percentage plaque burden, average plaque size and number of plaques. Immunohistochemical labelling with the anti-Aβ antibody 4G8 revealed a significant reduction in percentage plaque burden (Figure 6A,D), average plaque size (Figure 6B,E) and number of plaques (Figure 6C,F) in both the neocortex and the hippocampus of NIr-treated APP/PS1 mice relative to untreated APP/PS1 controls. Percentage plaque burden was reduced by over 40% in the neocortex (Figure 6A; *P* < 0.001) and over 70% in the hippocampus (Figure 6D; *P* < 0.01), average plaque size was reduced 25% in the neocortex (Figure 6B) and 30% in the hippocampus (Figure 6E), and the number of plaques was reduced by over 20% in the neocortex (Figure 6C) and by over 55% in the hippocampus (Figure 6F; all *P* < 0.05).

The photomicrographs of the 4G8 immunoreactivity in Figure 6 reflect the quantitative data described earlier. The WT brain is free of plaques (Figure 6H,K); many 4G8^+^ plaques (arrows) are present in the neocortex (Figure 6I) and the hippocampus (Figure 6L) of untreated APP/PS1 mice, and fewer plaques are present in NIr-treated APP/PS1 mice (Figure 6J,M). Comparable immunolabelling was achieved using the 6E10 anti-Aβ antibody (data not shown).

**Figure 7:**
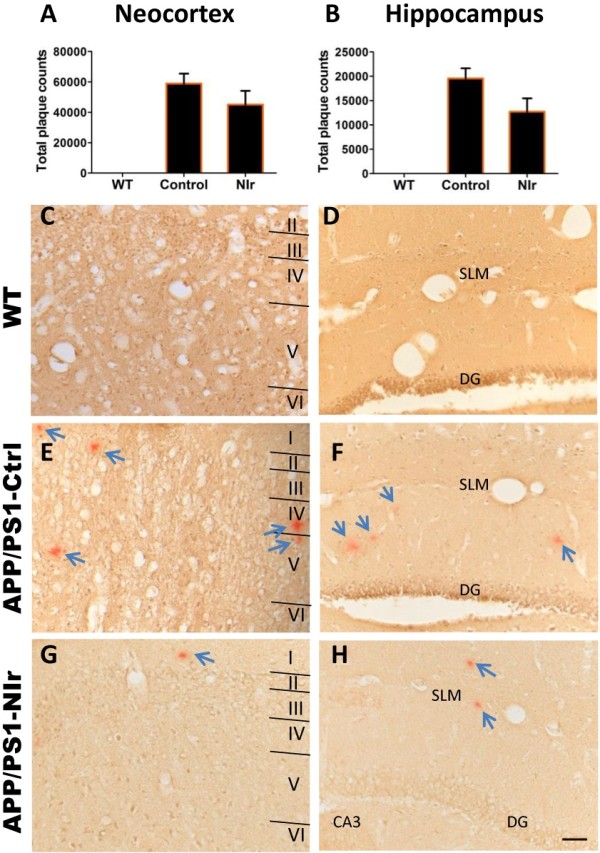
**Effect of near-infrared light on Congo red-positive plaque numbers in APP/PS1 mice. (A), (B)** Quantification of Congo red-positive plaque counts in the neocortex **(A)** and hippocampus **(B)**. All error bars indicate standard error of the mean. **(C), (D), (E), (F), (G), (H)** Representative micrographs showing Congo red staining of plaques in the neocortex **(C, E, G)** and hippocampus **(D, F, H)**. Arrows indicate plaques. Scale bar = 50 μm; scale in **(H)** applies to all other micrographs. DG, dentate gyrus of hippocampus; NIr, near-infrared light; SLM, stratum lacunosum moleculare; WT, wildtype.

A similar but less pronounced trend was observed when staining with Congo red (Figure 7), which stains only mature plaques. Mean counts of plaques in the neocortex (Figure 7A) and the hippocampus (Figure 7B) of NIr-treated APP/PS1 brains were lower than mean counts in untreated APP/PS1 brains (reductions >30%). However, the differences did not reach statistical significance; given the findings described above with the 4G8 and 6E10 anti-Aβ antibodies, this suggests that NIr may have greatest effect on recently formed Aβ deposits. The micrographs in Figure 7 show that mature plaques were absent from the WT brain (Figure 7C,D) but were present in the neocortex (Figure 7E) and hippocampus (Figure 7F) of untreated APP/PS1 brains. There appeared to be fewer plaques in the NIr-treated APP/PS1 brains (Figure 7G,H).

## Discussion

Using two mouse models with distinct AD-related pathologies (tau pathology in K3, amyloid pathology in APP/PS1), we report evidence that NIr treatment can mitigate the pathology characteristic of AD as well as reduce oxidative stress and restore mitochondrial function in brain regions affected early in the disease. Further, the extent of mitigation – to levels less than at the start of treatment – suggests that NIr can reverse some elements of AD-related pathology.

The present results add to our previous findings of NIr-induced neuroprotection in models of toxin-induced acute neurodegeneration (that is, MPTP-induced parkinsonism). When incorporated into the growing body of evidence that NIr can also protect against CNS damage in models of stroke, traumatic brain injury and retinal degeneration [9-12,36], the findings provide a basis for trialling NIr treatment as a strategy for protection against neurodegeneration from a range of causes. Present evidence is based on the use of multiple methods, immunohistochemical and histological, to demonstrate pathological features (for example, 4G8 antibody labelling and Congo red staining for amyloid plaques, AT8 antibody labelling and Bielschowsky silver staining for NFTs).

### Relationship to previous studies

The present study focused on pathological features considered characteristic of AD, as well as on signs of cellular damage (for example, oxidative stress, mitochondrial dysfunction) that have been demonstrated in AD and in animal models [2-4]. Our observations in the K3 strain add to previous studies by providing the first evidence in this strain of extensive oxidative damage and mitochondrial dysfunction [27].

Our findings are consistent with previous reports of the effects of red to infrared light on AD pathology in animal models. De Taboada and colleagues assessed the capacity of 808 nm laser-sourced infrared radiation, delivered three times per week over 6 months, to reduce pathology in an APP transgenic model of Aβ amyloidosis [17]. Treatment led to a reduction in plaque number, amyloid load and inflammatory markers, an increase in ATP levels and mitochondrial function, and mitigation of behavioural deficits. De Taboada and colleagues commenced treatment at 3 months of age, before the expected onset of amyloid pathology and cognitive effects. Similarly, Grillo and colleagues reported that 1,072 nm infrared light, applied 4 days per week for 5 months, reduces AD-related pathology in another APP/PS1 transgenic mouse model (TASTPM) [16]. These investigators also initiated light treatment before the onset of pathology, at 2 months of age. Both studies thus provide evidence that infrared radiation can slow the progression of cerebral degeneration in these models. The present results confirm these observations, in two distinct transgenic strains; they also confirm that the wound-healing and neuroprotective effects of red-infrared length do not vary qualitatively with wavelength, over a wide range.

### Evidence of reversal of pathology

Previous reports have described the natural history of the K3 [21,22] and APP/PS1 transgenic models [24,37]. Based on these previous reports and our own baseline data (Figure 1), significant brain pathology and functional deficits are present in both models at the ages when we commenced treatment. Our results therefore suggest that significant reversal of pathology has been induced by the NIr treatment. This has implications for clinical practice, where most patients are not diagnosed until pathogenic mechanisms have already been initiated and resultant neurologic symptoms manifest [15,27].

This evidence that AD-related neuropathology can be transient – appear then disappear – is not novel. Garcia-Alloza and colleagues described evidence of the transient deposition of Aβ, including the formation of plaque-like structures, in a transgenic model of Aβ deposition [24]. Reversal of such pathology, by interventions such as NIr treatment, may therefore be possible. However our results suggest that reversal may also be limited to recently formed, immature plaques, as we observed a significant NIr-induced reduction in immunolabelling with the 4G8 and 6E10 antibodies but no significant difference in Congo red staining. Because the 4G8 and 6E10 antibodies recognise various forms of Aβ, while Congo red stains only mature, compacted plaques, a reasonable deduction is that NIr treatment reduces only the transient, recently formed Aβ deposits, with no substantial effect on mature plaques. As there is still no consensus as to the pathogenic roles of different forms of Aβ, it is unclear how this might impact on the therapeutic potential of NIr in a clinical setting.

### Mechanisms

The mechanisms underlying the neuroprotective actions of red to infrared light are not completely understood. There is considerable evidence that NIr photobiomodulation enhances mitochondrial function and ATP synthesis by activating photoacceptors such as COX and increasing electron transfer in the respiratory chain, while also reducing harmful reactive oxygen species [38-40]. NIr photobiomodulation could also upregulate protective factors such as nerve growth factor and vascular endothelial growth factor [41,42] and mesenchymal stem cells [43] that could target specific areas of degeneration.

The ability of NIr to reduce the expression of hyperphosphorylated tau, which in turn reduces oxidative stress [44], may be key to its neuroprotective effect. Oxidative stress and free radicals increase the severity of cerebrovascular lesions [45,46], mitochondrial dysfunction [4,47], oligomerisation of Aβ [5,48] and tauopathies and cell death [48,49] in AD. Considering the brain’s high consumption of oxygen and consequent susceptibility to oxidative stress, mitigating such stressors would probably have a pronounced protective effect [50].

## Conclusions

Overall, our results in two transgenic mouse models with existing AD-related pathology suggest that low-energy NIr treatment can reduce characteristic pathology, oxidative stress and mitochondrial dysfunction in susceptible regions of the brain. These results, when taken together with those in other models of neurodegeneration, strengthen the notion that NIr is a viable neuroprotective treatment for a range of neurodegenerative conditions. We believe this growing body of work provides the impetus to begin trialling NIr treatment as a broad-based therapy for AD and other neurodegenerations.

## Abbreviations

Aβ: Amyloid-beta; AD: Alzheimer’s disease; APP: Amyloid beta precursor protein gene; COX: Cytochrome *c* oxidase; 4-HNE: 4-hydroxynonenal; LED: Light-emitting diode; NFT: Neurofibrillary tangle; NIr: Near-infrared light; 8-OHDG: 8-hydroxy-2′-deoxyguanosine; PS1: Presenilin 1; WT: Wildtype.

## Competing interests

The authors declare that they have no competing interests.

## Authors’ contributions

SP undertook the bulk of the experimental work and analysis and wrote the manuscript. DMJ and JM were involved with the analysis of the data and the writing of the manuscript. CN was involved with genotyping and treating the animals. JS was involved in conceiving and designing the study and the writing of the manuscript. All authors read and approved the final manuscript.
